# Hepatopulmonary syndrome secondary to metabolic associated fatty liver disease in childhood — novel treatment with growth hormone replacement therapy: a case report and systematic review of literature

**DOI:** 10.3389/fendo.2024.1407686

**Published:** 2024-10-22

**Authors:** Yunsoo Choe, Yun Jeong Lee, Young Ah Lee, Jae Sung Ko, Choong Ho Shin

**Affiliations:** ^1^ Department of Pediatrics, Hanyang University Guri Hospital, Guri, Republic of Korea; ^2^ Department of Pediatrics, Hanyang University College of Medicine, Seoul, Republic of Korea; ^3^ Department of Pediatrics, Seoul National University College of Medicine, Seoul, Republic of Korea

**Keywords:** metabolic associated fatty liver disease, hepatopulmonary syndrome, craniopharyngioma, hypopituitarism, growth hormone

## Abstract

**Objective:**

Hepatopulmonary syndrome (HPS) is a rare complication of metabolic associated fatty liver disease (MAFLD) occurring subsequent to hypopituitarism, often developing after resection of hypothalamic or pituitary tumors. The aim of this study is to report an illustrative case of an HPS patient who was successfully treated with growth hormone replacement therapy, without liver transplantation which is conventionally regarded as the only treatment option. Additionally, we conducted a comprehensive review of published case reports of HPS in the pediatric population.

**Methods:**

We systematically searched literature databases to identify case reports and case series of HPS associated with hypopituitarism diagnosed in childhood. The search included MEDLINE/PubMed, Scopus, Embase, and Google Scholar from 1990 to 2023. The review process adhered to the PRISMA checklist for comprehensive reporting and methodological transparency.

**Results:**

An 18-year-old female, who had been followed up for MAFLD after craniopharyngioma resection, presented with cyanosis and progressive dyspnea. She was diagnosed with severe degree of HPS. The patient began treatment with recombinant human growth hormone, leading to a significant improvement in respiratory symptoms within 3 months, and normalization of lung shunt ratio after 6 months of therapy. In our systematic review, nine patients from nine studies across six countries were identified. The median age at diagnosis of hypopituitarism was 10.5 years (range 1–16 years), and HPS was diagnosed at a median interval of 7 years later (range 0–26 years). Half of the patients had not received growth hormone therapy after being diagnosed with hypopituitarism, which subsequently led to the diagnosis of HPS. Three patients underwent liver transplantation, but non-alcoholic steatohepatitis recurred in all cases. Six patients were successfully treated with growth hormone replacement therapy without undergoing liver transplantation.

**Conclusions:**

HPS can occur in pediatric patients with MAFLD who have undergone resection of the tumor in the hypothalamus or pituitary gland. Our findings suggest that growth hormone replacement therapy can be a possible alternative to liver transplantation for HPS patients. However, further investigations need to be performed to validate the efficacy of growth hormone treatment in different causes of HPS cases.

## 1 Introduction

Hepatopulmonary syndrome (HPS) is a rare complication of metabolic associated fatty liver disease (MAFLD) which can occur after the surgical resection of hypothalamic or pituitary tumors. Characterized by arterial oxygenation defects due to intrapulmonary vasodilatations (1), HPS presents significant diagnostic and therapeutic challenges. Currently, liver transplantation (LT) is the only established effective treatment, with no medical therapies proven useful to date (2, 3). Since patients in the early phase of HPS can be frequently asymptomatic, the disease is often under-recognized, resulting in delayed diagnosis (4). However, considering its significant morbidity and mortality, prompt diagnosis and management are necessary.

Patients with hypopituitarism may be susceptible to developing HPS (5). This may be attributed to the dysfunction of the hypothalamus or pituitary gland, leading to the development of MAFLD and non-alcoholic steatohepatitis (NASH) (6), a predisposing factor for HPS. Craniopharyngioma, the most common suprasellar tumor in children, can induce hypothalamic injury and hormonal dysregulation and is thus often associated with hypothalamic obesity and development of MAFLD (7). Although few cases of HPS associated with hypopituitarism secondary to craniopharyngioma resection have been reported (5, 8, 9), treatment courses in this population have rarely been investigated.

In this comprehensive review, we analyzed previous reports of pediatric HPS cases following neurosurgical procedures for pituitary or hypothalamic lesions, along with a detailed case report of HPS children successfully treated with growth hormone replacement therapy. The aim of this review and case report is to provide clinicians and surgeons with information regarding the extended clinical course of pediatric patients undergoing pituitary surgery and suggest non-surgical alternatives for the management of HPS.

## 2 Materials and methods

### 2.1 Study design

We present a case of HPS that occurred after surgical resection of craniopharyngioma in childhood. Written informed consent to participate in this study was provided by the patient and the parents of the patient. We also conducted a systematic review of the literature to identify previous case reports of pediatric HPS patients. Our review process adhered to the protocols outlined in the Preferred Reporting Items for Systematic Reviews and Meta-Analyses 2020 flow diagram.

### 2.2 Search strategy and eligibility criteria

We searched MEDLINE/PubMed, Scopus, Embase, and Google Scholar databases for case reports and case series published in a peer-reviewed journal between 1990 and 2023. An exploratory search was conducted between October 13th and October 31st, 2023 using the following search terms: (Hepatopulmonary syndrome OR HPS OR Hepatopulmonary shunt OR Intrapulmonary shunt) AND (Hypopituitarism OR Pituitary dysfunction OR growth hormone deficiency). The inclusion criteria were (1) case reports or case series of HPS that developed following hypopituitarism and (2) reports written in English. The exclusion criteria were (1) reviews, conference or congress abstracts, commentaries, letters to editors, or any unpublished data, (2) documents not in English, and (3) did not provide information on the treatment course. This systematic review was conducted only on case reports and case series, as there have been no randomized controlled trials, prospective cohort studies, or case-controlled studies available. The search, identification, and categorization processes were performed independently by two reviewers (Y.C and C.H.S).

### 2.3 Data extraction and study quality evaluation

Data on the name of authors, time of publication, gender of the case, age at diagnosis of hypopituitarism and diagnosis of HPS, and cause of hypopituitarism were extracted from the case reports and case series. Treatment modality and duration of rHGH before onset of HPS, and clinical outcome were also collected. Study quality was evaluated using the Newcastle-Ottawa Scale for Assessing the Quality of Non-randomized Studies.

## 3 Results

### 3.1 Illustrative case

An 18-year-old girl was referred to a tertiary care center in December 2020 for the evaluation of cyanosis for 10 months and progressive dyspnea for 3 months. She presented with cyanotic lips without finger clubbing or hepatosplenomegaly. Her oxygen saturation (SpO2) was 77% on room air.

She had received trans-sphenoidal resection surgery for a craniopharyngioma at 12 years of age and was started on levothyroxine, hydrocortisone, and desmopressin. At 10 months postoperatively, she was diagnosed with GH deficiency after a combined anterior pituitary function test (**Table 1**), and recombinant human GH (rHGH) was administered. However, 2 months later, tumor recurrence was detected in the posterior aspect of the sella turcica, leading to discontinuation of rHGH and gamma knife surgery (GKS). Due to persistent tumor-like lesions observed in repeated MRI examinations following GKS, the administration of rHGH replacement therapy was deferred. She was followed up for hypopituitarism and moderate-to-severe degree of MAFLD.

**Table 1:** Results of combined pituitary function tests of the patient 10 months after trans-sphenoidal resection of craniopharyngioma and at the time of diagnosis of hepatopulmonary syndrome.

Hormone	After surgery		At diagnosis of hepatopulmonary syndrome	
	Basal	Peak	Basal	Peak
Human growth hormone (ng/mL)	0.05 (0.3–8.7)	0.09 (>10)	0.08 (0–10)	0.05 (>10)
Luteinizing hormone (mIU/mL)	0.5 (1–12)	1.0 (>10)	0.6 (1–12)	1.2 (>10)
Follicle-stimulating hormone (mIU/mL)	<0.5 (2–13)	<0.5 (>2)	<0.5 (2–13)	0.8 (>2)
Estradiol (pg/mL)	29 (30–120)		3 (30–120)	
Adrenocorticotropic hormone (pg/mL)	5.2 (0–60)		13.9 (0–60)	
Cortisol (μg/dL)	0.5 (3.7–19.4)	1.3 (>18)	0.2 (3.7–19.4)	0.3 (>18)
Thyroid stimulating hormone (μIU/mL)	0.01 (0.35–4.94)		1.10 (0.4–5.0)	
Free thyroxine (ng/dL)	0.87 (0.70–1.48)		0.81 (0.7–1.8)	
Triiodothyronine (ng/dL)	150.5 (58–159)		122 (87–184)	

Reference ranges of each hormone are expressed in parenthesis.

At presentation, her height, body weight, and body mass index were 160.2 cm (−0.14 standard deviation score [SDS]), 63.2 kg (1.19 SDS), and 24.6 kg/m^2^ (1.37 SDS), respectively. Arterial blood gases on room air revealed a pH of 7.416, PaCO2 of 31.5 mmHg, and PaO2 of 48.2 mmHg with increased alveolar-arterial oxygen pressure difference (62.4 mmHg, normal range 5–10). Laboratory examination revealed slightly elevated liver enzyme level (aspartate transaminase, 46 IU/mL; alanine transaminase, 20 IU/mL; reference range 0–40), hyperbilirubinemia (total bilirubin concentration, 1.6 mg/dL; reference range 0.2–1.2), thrombocytopenia (platelet count of 93 × 10^3^/μL), and prolonged prothrombin time (1.46 international normalized ratio, reference range 0.8–1.2), suggesting liver dysfunction. The fibrosis 4 (FIB-4) index [= age (years) × AST (U/L)/platelet count (10^9^/L) × √ALT (U/L)] was 1.99, indicating an intermediate risk of advanced liver fibrosis. Combined anterior pituitary hormone test results indicated multiple pituitary hormone deficiencies (**Table 1**).

**Figure 1:**
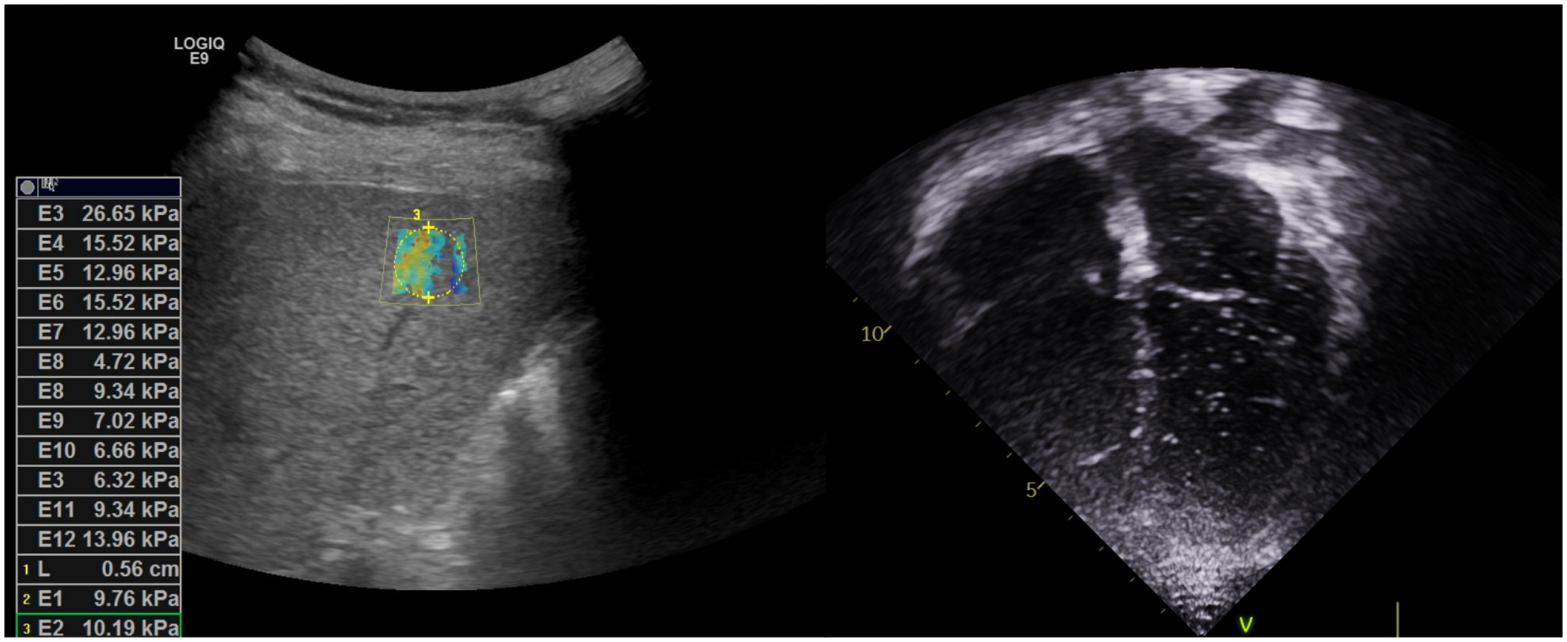
Left: Liver elastography revealed slightly coarse and increased liver parenchymal echogenicity, and increased liver stiffness index. Right: Contrast-enhanced echocardiography with agitated saline revealed microbubbles appearing in the left ventricle after three cardiac cycles following the appearance of bubbles in the right ventricle, indicating the presence of right to left intrapulmonary shunt.

**Figure 2:**
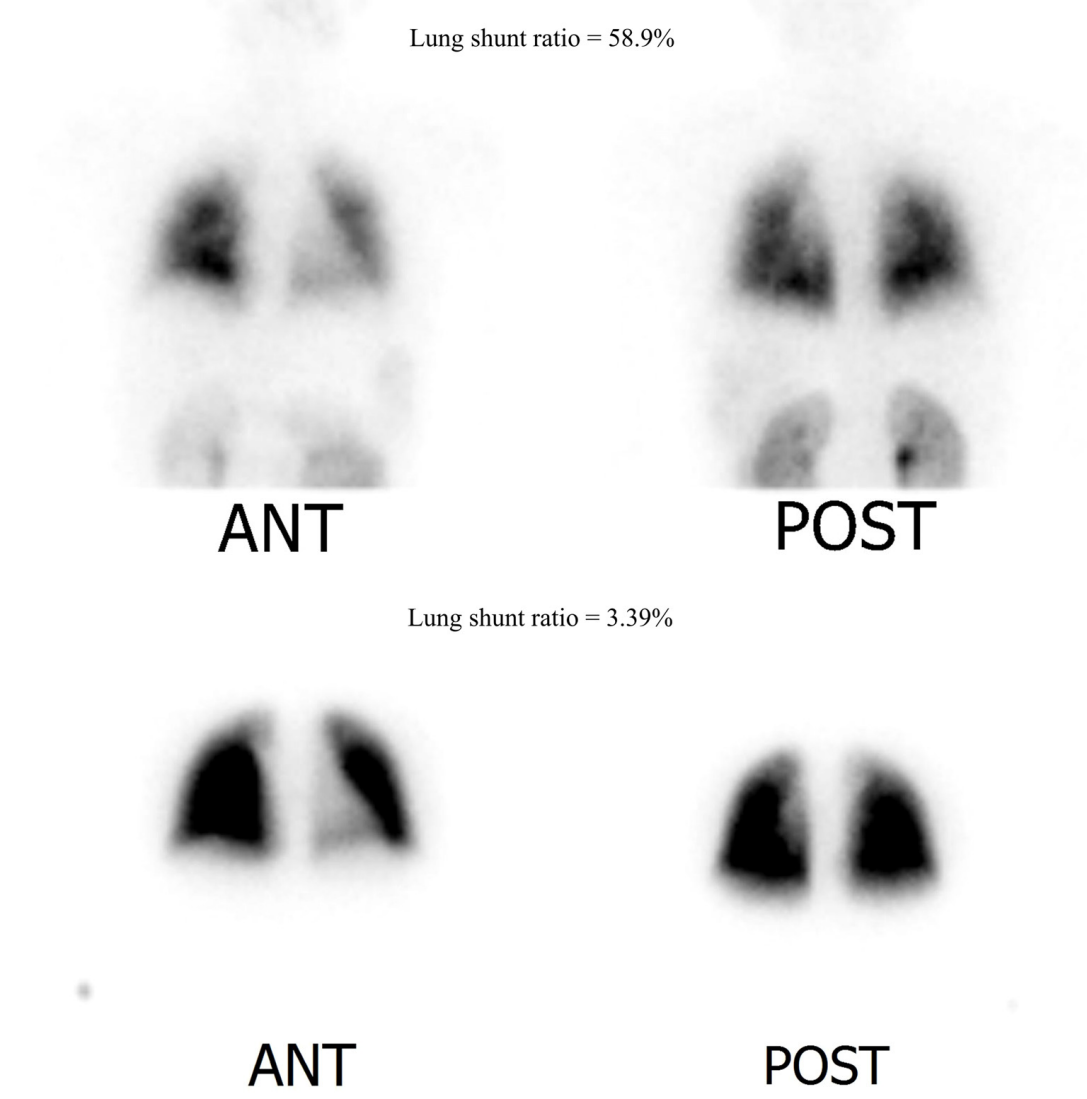
Lung perfusion scan image (normal range < 6%). Upper row: Initial lung perfusion scan indicating hepatopulmonary shunt (lung shunt ratio = 58.9%). Lower row: Follow-up lung perfusion scan after recombinant growth hormone replacement treatment for 6 months. Hepatopulmonary shunt improved with a lung shunt ratio of 3.39%.

Abdominal computed tomography scan revealed undulation of the liver surface and blunting of the liver contour, with a relatively small right liver volume compared to the left liver volume and splenomegaly. Elastography revealed increased liver stiffness with a mean elasticity of 12.44 kPa (**Figure 1**). Contrast-enhanced echocardiography (**Figure 1**) and radioactive lung perfusion scan (**Figure 2**) confirmed an intrapulmonary shunt, leading to the diagnosis of very severe HPS (1).

Oxygen therapy was initiated, and the patient was registered for LT. rHGH replacement therapy was initiated with incremental doses, leading to improved oxygenation and a reduced shunt ratio. After 3 months, her respiratory symptoms improved significantly, with improved SpO2 mostly remaining in the 90s, with occasional reduction to 80s without oxygen. The patient was able to climb the 4th floor unassisted. After 6 months, the shunt ratio improved to 3.39% on repeated lung perfusion scans (**Figure 2**), and the patient was excluded from the waiting list for LT.

One year after HPS diagnosis, her respiratory symptoms resolved, and SpO2 remained above 90% without oxygen support. The FIB-4 index was improved to 0.81, and liver stiffness was also significantly improved to 8.73 kPa in elastography. Her brain MRI revealed no changes in the previously observed abnormalities.

### 3.2 Results of the systematic review

**Figure 3:**
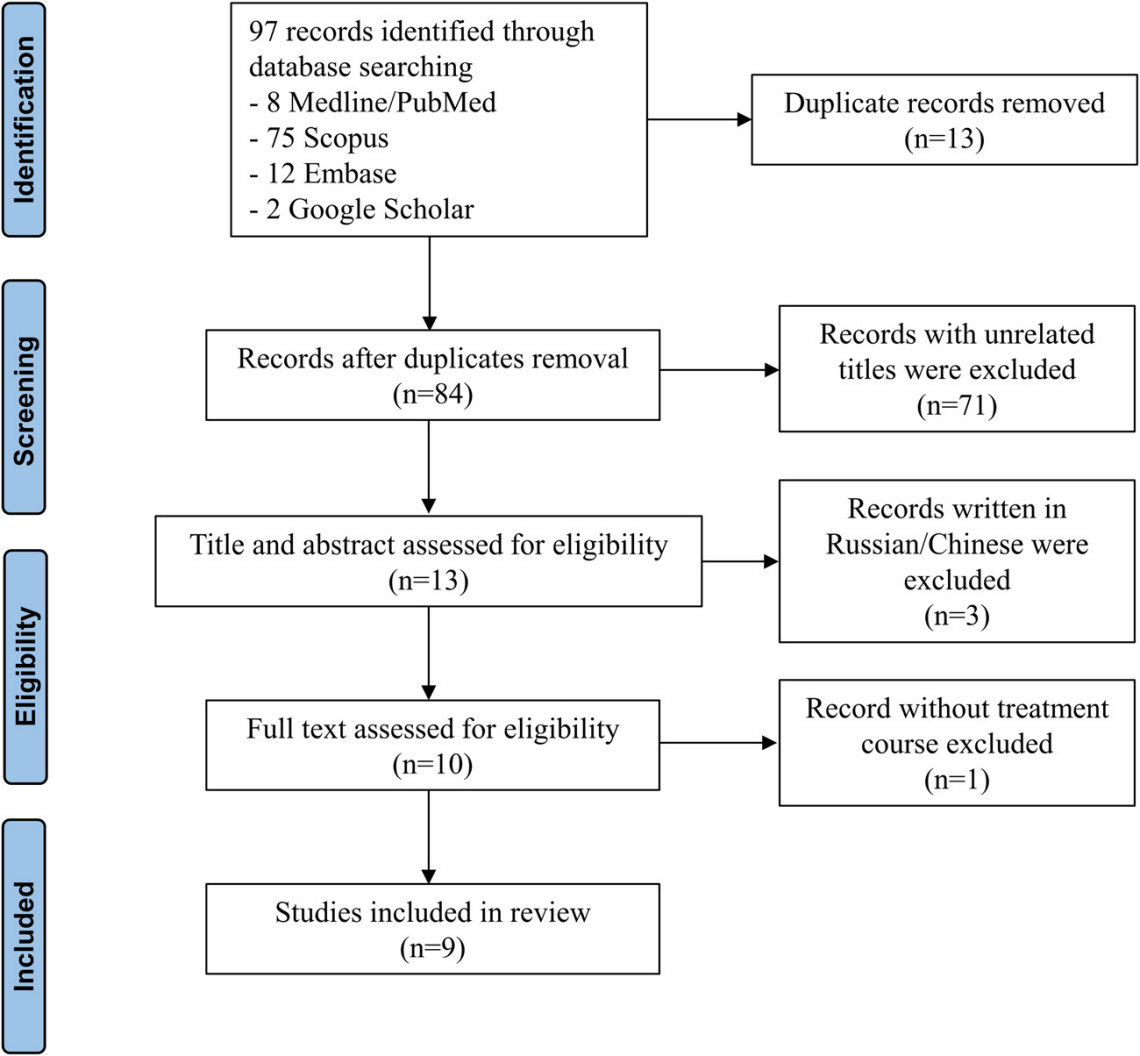
PRISMA flow diagram of the literature review.

We identified a total of 84 non-duplicated records, of which 71 reports were excluded because their titles were unrelated, and 3 reports were excluded because they were written in Russian or Chinese (**Figure 3**). After excluding 1 record that did not include the treatment course, 9 articles (2–6, 8–11) were finally included. All records were case reports, which were evaluated as low levels of evidence (**Table 2**).

**Table 2:** Quality of the studies evaluated by the Newcastle-Ottawa Quality Assessment for Assessing Non-randomized Studies.

References	Selection				Comparability	Outcome/Exposure			Quality category
	Representativeness of the exposed cohort	Selection of the non-exposed cohort	Ascertainment of exposure	Outcome of interest not present at start of study	Comparability of cohorts on the basis of the design or analysis	Ascertainment of outcome	Adequacy of duration of follow-up	Adequacy of completeness of follow-up	
Zhang et al., 2022 (2)	★		★			★	★	★	Poor
Alabsawy et al., 2021 (5)	★		★			★	★	★	Poor
Ji et al., 2021 (6)	★		★			★	★	★	Poor
Torii et al., 2018 (10)	★		★			★	★	★	Poor
Jung et al., 2018 (3)	★		★			★	★	★	Poor
Fujio et al., 2015 (8)	★		★			★	★	★	Poor
Justino et al., 2010 (9)	★		★			★	★	★	Poor
Jankowska et al., 2007 (4)	★		★			★	★	★	Poor
Jonas et al., 2005 (11)	★		★			★	★	★	Poor

Solid star represents one score, and the blank represents zero score.

### 3.3 Characteristics and treatment course of HPS in the literature

The clinical characteristics of the included case reports are described in **Table 3**. Of the included 9 cases, 2 cases were reported from USA (9, 11), 2 from Japan (8, 10), 2 from China (2, 6), 1 from Korea (3), 1 from Poland (4), and 1 from United Kingdom (5). Among 10 patients, including our case, 4 developed hypopituitarism after the resection of craniopharyngioma (2–4). Other patients were reported to have pituitary stalk interruption syndrome (6), pituitary stalk rupture (10), or after surgical resection of mature teratoma/germinoma (8), Langerhans cell histiocytosis of the sella turcica (9), and non-neoplastic hypothalamic tumor (11), respectively. Patients were diagnosed with hypopituitarism at a median age of 10.5 years (range 1–16 years), followed by the diagnosis of HPS at a median interval of 7 years (range 0–26 years). Five out of 10 patients (50%) never received rHGH replacement therapy after being diagnosed with hypopituitarism (2–4, 9, 11). Other patients, including our case, did not receive rHGH for most of the duration between the diagnosis of hypopituitarism and HPS (5, 6, 10). Three patients underwent LT (30.0%) (4, 8, 11), while NASH recurred in all cases, with recurrence times noted as 5 years (8), 6 months (4), and 2 months (11). Notably, one of these patients was reported to have been stable for 5 years after the initiation of rHGH treatment. Six patients (60.0%) reported improvement of HPS with rHGH treatment. Among them, Zhang et al (2) and Ji et al (6) documented the fastest improvement of respiratory symptoms in just 1 month, with shunt ratio normalization achieved after 12 and 14 months, respectively. In our case, the patient reported respiratory symptom improvement after 3 months of rHGH replacement, and shunt ratio was rapidly normalized within 6 months. There has been one reported case of HPS improvement without undergoing either LT or rHGH therapy; Justino et al (9) demonstrated the resolution of HPS within 4 months exclusively through weight reduction and calorie restriction. However, 2 months later, the patient developed porto-pulmonary hypertension and received pulmonary vasodilator treatment, resulting in a stable clinical course.

**Table 3:** Clinical characteristics and treatment courses in patients with hepatopulmonary syndrome caused by hypopituitarism.

References	Sex	Age at diagnosis of hypopituitarism (years)	Age at diagnosis of HPS (years)	Cause of hypopituitarism	rHGH duration before HPS onset	Initial treatment	Treatment course
This case	F	12	18	Craniopharyngioma	2 months	rHGH	Respiratory symptoms improved in 3 months, and shunt ratio returned to the normal range in 6 months
Zhang et al., 2022 (2)	F	7	13	Craniopharyngioma	No	rHGH	Respiratory symptoms improved after 1 month, and liver function and oxygenation improved after 1 year.
Alabsawy et al., 2021 (5)	M	1	27	ND	17 years	rHGH	Awaiting a donor for LT
Ji et al., 2021 (6)	M	10	29	Pituitary stalk interruption syndrome	5 years	rHGH	Respiratory symptoms improved in 1 month, and shunt ratio returned to the normal range after 14 months of rHGH treatment.
Torii et al., 2018 (10)	M	2	23	Pituitary stalk rupture	6 years	rHGH	NASH improved in follow-up biopsy performed after 5 months.
Jung et al., 2018 (3)	F	11	19	Craniopharyngioma	ND	rHGH	Awaiting a donor for LT
Fujio et al., 2015 (8)	M	11	15	Mature teratoma/germinoma	No	LT	NASH recurred 5 years after LT, followed by the initiation of rHGH treatment. The patient has been stable for the next 5 years.
Justino et al., 2010 (9)	F	14	17	Langerhans cell histiocytosis of the sellar turcica	No	weight reduction with calorie restriction	Resolution of HPS within the first 4 months but progressed to porto-pulmonary hypertension after 2 months. The patient has remained stable with pulmonary vasodilator treatment.
Jankowska et al., 2007 (4)	M	6	12	Craniopharyngioma	No	LT	NASH recurred in 6 months.
Jonas et al., 2005 (11)	M	16	16	Non-neoplastic hypothalamic tumor	No	rHGH	LT was performed 7 months after presentation and rHGH treatment. However, NASH recurred 2 months post-LT.

HPS, hepatopulmonary syndrome; rHGH, recombinant human growth hormone; LT, liver transplantation; NASH, non-alcoholic steatohepatitis; ND, no data; F, female; M, male.

## 4 Discussion

In this pediatric case of HPS secondary to hypopituitarism following craniopharyngioma resection, we successfully treated the patient with GH therapy, eliminating the need for LT, which is the only therapeutic option for HPS.

Initially described by Kennedy and Knudson in 1977 (12), HPS primarily manifests as hypoxemia due to impaired gas exchange resulting from alterations in the pulmonary microvasculature (1). The vasodilatation of pulmonary vessels and the formation of direct arterio-venous shunts contribute to ventilation-perfusion mismatch, while alveolar dysfunction by impaired alveolar integrity further worsens gas exchange (1, 13). HPS has been reported to be associated with hypothalamic dysfunction or hypopituitarism (**Table 2**) (2–6, 8–11), with some cases linked to the resection of tumors located in the hypothalamus or sellar lesion (2–4, 8, 9) including craniopharyngioma.

Craniopharyngioma is a benign epithelial tumor of dysembryogenic origin arising suprasellar area and often involving the hypothalamus (14). Resection of the tumor frequently leads to hypopituitarism, which results in various endocrine disorders accompanied with hormonal imbalances due to surrounding tissue injuries following surgery. Particularly, the development of insulin resistance and hypothalamic obesity can increase intrahepatic fat accumulation. Additionally, GH deficiency can also lead to significant fat accumulation in the viscera and liver, contributing to the development of NASH and MAFLD, along with metabolic syndrome.

In addition to GH deficiency, impaired appetite regulation and compromised energy expenditure may contribute to MAFLD development in patients with hypothalamic dysfunction (14). Leptin, a cytokine produced exclusively in adipose tissue, functions in the hypothalamus to reduce food intake and increase energy expenditure (15). Therefore, damage to the hypothalamus during cranial tumor surgery may likely disrupt the function of leptin, resulting in hyperphagia and reduced metabolic rate. Moreover, leptin levels are significantly increased in patients with hypopituitarism, indicating a “leptin resistance” state (15, 16). Leptin acts as a fibrogenic cytokine and plays a key regulatory role in the progression of fibrosis and inflammation in chronic liver disease, including MAFLD and NASH (15).

Although LT remains the primary treatment for HPS, some researchers have reported improvements in respiratory symptoms (2, 6, 10) with rHGH therapy, which has enabled the patients to avoid LT. GH has also been reported as effective in reducing hepatic fat content in patients with MAFLD (17, 18). This can be explained by the role of GH in directly inhibiting lipogenesis and indirectly activating hormone-sensitive lipase, which reduces adipose deposition in the liver (19). Additionally, insulin-like growth factor-1 alleviates liver cirrhosis by inducing cell senescence and inactivating hepatic stellate cells (20). Altogether, the GH/insulin-like growth factor-1 axis plays an essential role in preventing hepatic steatosis aggravation in fatty liver disease, which may ameliorate the respiratory symptoms in HPS (19, 21).

To date, investigational medical therapies have been used to treat HPS; however, no significant advantages have been established over LT. Vasodilators, such as pentoxifylline, inhaled nitric oxide (NO), and iloprost, as well as inhibitors of angiogenesis including octreotide or sorafenib, have demonstrated no definite evidence of improving oxygenation in HPS (13). The use of NO synthase inhibitors and pulmonary vasoconstrictors has also been attempted, but no obvious benefits have been demonstrated (22). Although the favorable effect of GH in improving oxygenation in HPS has only been observed in those secondary to hypopituitarism (2, 3, 5, 6, 10), it is worthwhile to explore the potential effectiveness of GH in patients with HPS of alternative etiologies, considering recent reports indicating that GH replacement therapy may have a positive impact on non-alcoholic fatty liver disease (21). Not only pediatric hypopituitarism patients but also adult patients with hypopituitarism, GH deficiency, obesity, and MAFLD are reported to have positive effects from GH replacement therapy (21). Furthermore, in patients with decompensated cirrhosis, GH treatment in conjunction with standard medical therapy has shown beneficial effects across various parameters, including skeletal muscle index, liver frailty index, and Child-Turcotte-Pugh score for end-stage liver disease (23). While GH may not be universally beneficial in all patients with liver damage, it appears promising as a therapeutic option for those with hepatic impairment.

Craniopharyngioma is historically benign but locally aggressive and prone to frequent recurrence (14). In our case, the patient only received 2 months of rHGH therapy before being diagnosed with HPS owing to tumor recurrence. Likewise, Mazerkina et al. (24) reported a case of hypopituitarism subsequent to craniopharyngioma resection in a patient who had never received rHGH treatment before or after HPS diagnosis because of concerns regarding recurrence, ultimately resulting in death. In HPS, the symptoms may not be severe in the initial phase, often leading to a delayed diagnosis. The severity of liver disease and the degree of hypoxemia do not necessarily correlate (25). While MAFLD occurs in 50% of patients with craniopharyngioma (15), HPS can occur in 4–30% of patients with chronic liver disease (1). Therefore, carefully monitoring patients who have had craniopharyngioma resection surgery for HPS occurrence and actively considering GH treatment in this population is important.

In our study, rHGH replacement therapy was effective in improving HPS, consistent with similar findings in some studies reported previously. However, due to limited number of cases, the absence of randomized controlled trials, and the retrospective nature of the analysis, generalizing these results to all HPS patients may be challenging. The efficacy of rHGH replacement therapy may vary depending on factors such as the age at onset of hypopituitarism, duration of hormonal replacement treatment before onset of HPS, comorbidities, and the patient’s general condition at the time of diagnosis. Therefore, caution is warranted when applying this study results to patients with HPS.

## 5 Conclusions

HPS may develop in patients who have undergone resection for tumors in the hypothalamus or pituitary gland, particularly in those not currently receiving GH treatment. While evidence from the literature is limited, rHGH replacement therapy emerges as a promising treatment modality for HPS secondary to hypopituitarism, demonstrating successful improvements in oxygenation and normalization of intrapulmonary shunt. However, further investigations are required to determine the effectiveness of GH in patients with HPS in other underlying etiologies.

## Data Availability

The original contributions presented in the study are included in the article/supplementary material. Further inquiries can be directed to the corresponding authors.
